# Lack of neuroinflammation in the HIV-1 transgenic rat: an [^18^F]-DPA714 PET imaging study

**DOI:** 10.1186/s12974-015-0390-9

**Published:** 2015-09-17

**Authors:** Dianne E. Lee, Xuyi Yue, Wael G. Ibrahim, Margaret R. Lentz, Kristin L. Peterson, Elaine M. Jagoda, Michael Kassiou, Dragan Maric, William C. Reid, Dima A. Hammoud

**Affiliations:** Center for Infectious Disease Imaging (CIDI), Radiology and Imaging Sciences, National Institutes of Health/Clinical Center, 10 Center Drive, Room 1C368, Bethesda, MD 20814-9692 USA; Department of Radiology, Washington University School of Medicine, St. Louis, MO USA; Molecular Imaging Program (MIP), National Cancer Institute (NCI), Bethesda, MD USA; Chemistry Department, The University of Sydney, Sydney, Australia; Division of Intermural Research (DIR), National Institute of Neurological Disorders and Stroke (NINDS), National Institutes of Health, Bethesda, MD USA

**Keywords:** HIV, Transgenic rat, Positron emission tomography, Neuroinflammation

## Abstract

**Background:**

HIV-associated neuroinflammation is believed to be a major contributing factor in the development of HIV-associated neurocognitive disorders (HAND). In this study, we used micropositron emission tomography (PET) imaging to quantify neuroinflammation in HIV-1 transgenic rat (Tg), a small animal model of HIV, known to develop neurological and behavioral problems.

**Methods:**

Dynamic [^18^F]DPA-714 PET imaging was performed in Tg and age-matched wild-type (WT) rats in three age groups: 3-, 9-, and 16-month-old animals. As a positive control for neuroinflammation, we performed unilateral intrastriatal injection of quinolinic acid (QA) in a separate group of WT rats. To confirm our findings, we performed multiplex immunofluorescent staining for Iba1 and we measured cytokine/chemokine levels in brain lysates of Tg and WT rats at different ages.

**Results:**

[^18^F]DPA-714 uptake in HIV-1 Tg rat brains was generally higher than in age-matched WT rats but this was not statistically significant in any age group. [^18^F]DPA-714 uptake in the QA-lesioned rats was significantly higher ipsilateral to the lesion compared to contralateral side indicating neuroinflammatory changes. Iba1 immunofluorescence showed no significant differences in microglial activation between the Tg and WT rats, while the QA-lesioned rats showed significant activation. Finally, cytokine/chemokine levels in brain lysates of the Tg rats and WT rats were not significantly different.

**Conclusion:**

Microglial activation might not be the primary mechanism for neuropathology in the HIV-1 Tg rats. Although [^18^F]DPA-714 is a good biomarker of neuroinflammation, it cannot be reliably used as an in vivo biomarker of neurodegeneration in the HIV-1 Tg rat.

**Electronic supplementary material:**

The online version of this article (doi:10.1186/s12974-015-0390-9) contains supplementary material, which is available to authorized users.

## Background

In the developed world where antiretroviral therapy (ART) is readily available, HIV/AIDS has been transformed from a once fatal to a chronic manageable disease, with markedly decreased mortality and morbidity. Along the same lines, the incidence of severe neurocognitive dysfunction decreased significantly [1]. The more subtle forms of neurocognitive dysfunction however became more prevalent, leading to gradual, but ultimately significant functional deterioration of otherwise virologically controlled HIV+ patients [2].

Among various factors, the contribution of neuroinflammation/microglial activation to neuronal damage in HIV is assumed to play a major role [3] based on multiple cell culture studies [4–7] as well as direct histological evaluation of brain tissues from untreated HIV or simian immunodeficiency virus-infected animals [8]. The hallmark of neuroinflammation is the activation of naturally quiescent resident microglial cells [9] resulting in the unregulated secretion of multiple neurotoxins and cytotoxins, ultimately resulting in neuronal damage leading to cell death [3, 10]. The ability to non-invasively monitor neuroinflammation is thus an important target in the diagnosis, prevention, and evaluation of treatment effect in many neurological diseases including neuro-HIV.

Imaging microglial activation as a surrogate marker for neuroinflammation can be done through the use of specific radiolabeled ligands targeting the translocator protein (TSPO, previously known as the peripheral benzodiazepine receptor (PBR)) [3], an 18 kD outer mitochondrial membrane receptor which is naturally expressed in small amounts in resting microglial cells. TSPO however gets significantly upregulated during microglial activation [9]. The prototype positron emission tomography (PET) TSPO ligand, [^11^C]PK11195, has been extensively used to image neuroinflammation in a variety of neurodegenerative diseases [11–16] but has long been criticized for inherent limitations such as high non-specific binding and high lipophilicity [17–20]. Newer higher affinity ligands for TSPO, such as DPA-713 [21], PBR-28 [22], CLINDE [23, 24], and DAA [25], among others, have been developed as a result.

In this study, we set out to validate one of the newly designed TSPO ligands, [^18^F]DPA-714, an ^18^F-labeled pyrazolopyrimidine, [19, 20, 26], as surrogate imaging biomarker of microglial activation (neuroinflammation), in vivo, in the brains of HIV-1 transgenic rats (Tg) compared to age-matched controls. We wanted to test the hypothesis that the Tg rat brain shows microglial activation which would potentially allow the use of [^18^F]DPA-714 PET in this animal model as a biomarker for the effectiveness of various neuroprotective/anti-inflammatory therapies.

## Methods

### Animals

Experiments were carried out in male Tg (F344/Hsd) in three different age groups (3, 9, and 16 month-old) and male age-matched wild-type control rats (F344) (WT) purchased from Harlan Inc. (Indianapolis, IN). The total sample of animals used for PET imaging, blood metabolism experiments, and cytokine/chemokine measurements included 28 Tg and 27 WT rats. All rats were housed in a temperate-controlled environment with a 12-h light/dark cycle. The animals were allowed free access to food and water. All procedures were conducted during the light cycle. The rats were acclimated to careful handling prior to testing to minimize stress. Animal care and all experimental procedures were approved by the Institutional Animal Care and Use Committee (ACUC) of the National Institutes of Health (NIH).

### QA surgical procedures

Three WT rats weighing 210 ± 114 g were used for intrastriatal injection of quinolinic acid (QA). The animals were anesthetized with 1 mg/kg ketamine and xylazine (10:1) cocktail (Sigma) and then placed in a stereotaxic apparatus (Stoelting Wood Dale, IL, USA). Unilateral stereotaxic injections of 150 nmol in 1 μl of QA (Sigma; dissolved in 0.1 M phosphate-buffered saline (PBS), pH 7.4) in the right striatum were made over 5 min using a 10 μl Hamilton syringe fitted with a micropump (Stoelting Wood Dale, IL, USA). The syringe was fitted with a 26-gauge needle at the following coordinates according to Paxinos and Watson (1998): anteroposterior (AP) +1 mm, mediolateral (ML) −3.0 mm, dorsoventral (DV) −4.5 mm, from the bregma. The injection syringe was left in place for an additional 5 min to allow the QA to diffuse from the needle tip and avoid backflow. After removing the needle, the skin was sutured and the animals were allowed to recover before being returned to their cages. The animals were given buprenorphine (0.03 mg/kg) intramuscularly and examined daily until PET imaging. PET imaging studies were conducted either 7 days post-op (*n* = 1) or 3 days post-op (*n* = 2). All animal procedures were performed in accordance with the NIH ACUC guidelines.

### Radiochemistry

Automated syntheses of [^18^F]DPA-714 were carried out using a slightly modified TRACERLab FX-FN module (GE Medical Systems, Germany). In brief, aqueous [18F]fluoride anions were sucked through the Chromafix® under vacuum. The trapped ^18^F-fluoride was eluted from the cartridge and transferred to the reaction vessel with an eluent solution containing K_2_CO_3_, acetonitrile, and Kryptofix-222. The reaction mixture was evaporated to dryness after addition. Tosylate substrate was then dissolved in dimethyl sulfoxide, and the mixture was transferred to the dry 18F-labeled KF-K222 complex and allowed to react at 165 °C for 5 min. On completion, the reaction mixture was diluted with semi-preparative HPLC solvent and passed through a Sep-Pak® light Alumina N cartridge (Waters Corporation, Milford, MA). The reaction vessel was rinsed with HPLC solvent and passed through the Sep-Pak® Alumina N cartridge by helium pressure. The combined crude solution collected in the collection flask was transferred to the HPLC injection loop and injected into a Phenomenex Luna 5 μ C18 semi-preparative reversed-phase HPLC column (250 × 10 mm), with a mobile phase of H_2_O and acetonitrile (55/45, *v*/*v*) at a flow rate of 4.0 ml/min. The retention time (tR) of [^18^F]DPA-714 was determined to be 19 min. The radioactive fraction corresponding to [^18^F]DPA-714 was collected into a dilution flask. The final formulation of the tracer was performed automatically using a Sep-Pak® Plus C18-based system. The diluted fraction collected from the dilution flask was passed through the C18 cartridge to fix the tracer. The cartridge was washed with deionized water (10 ml), and the purified tracer was finally recovered by elution of the C18 cartridge with the ethanol (1.5 ml) from the reservoir. Yield and specific activity were determined at this stage (25 ± 3 %, specific activity 41–107 GBq/μmol, *n* > 10). The excess ethanol was removed through rotary evaporation and concentrated to 250–350 μl. The tracer was delivered and diluted with PBS for final formulation and animal administration.

### [^18^F]DPA-714 blood metabolism

**Fig. 1:**
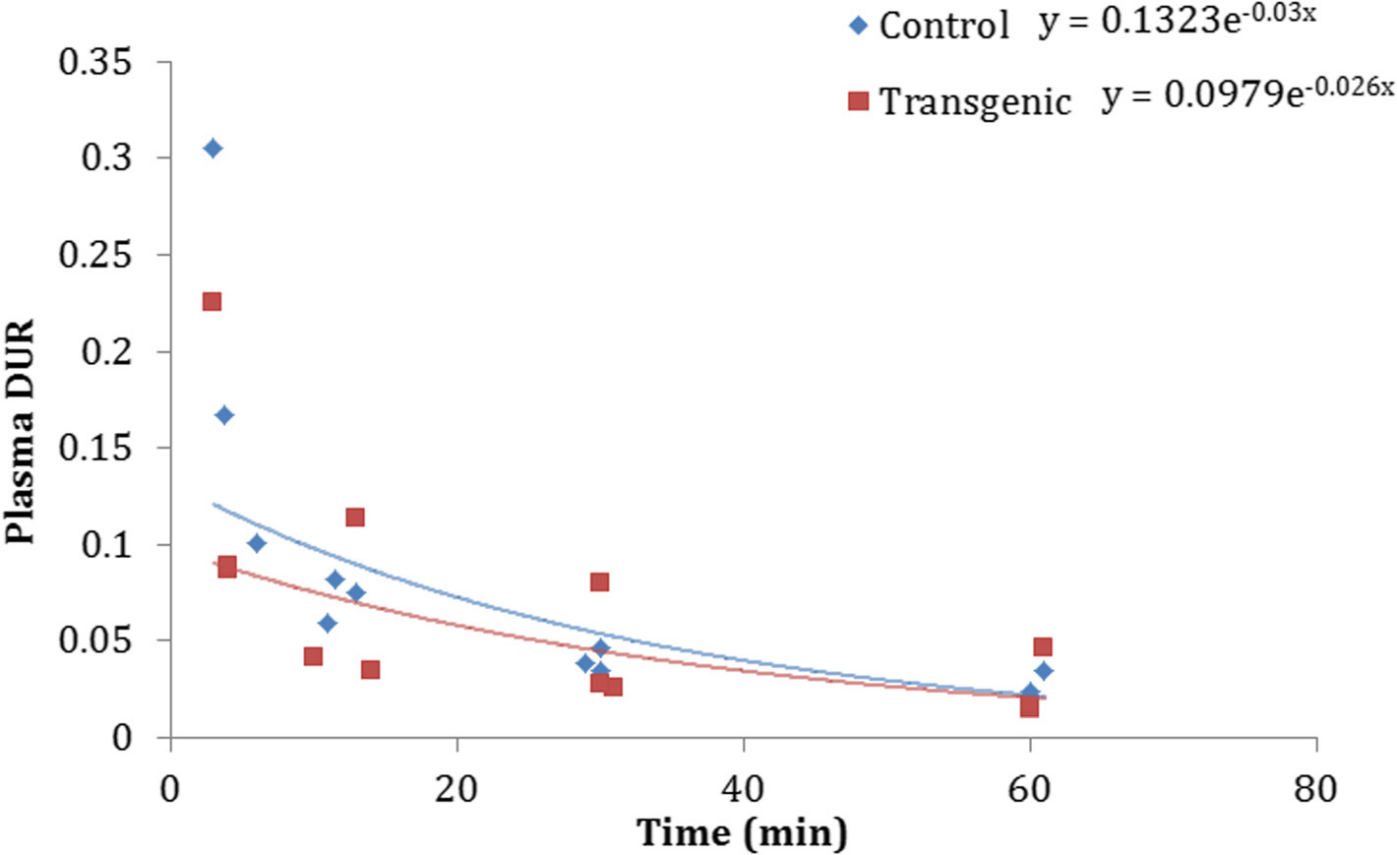
[^18^F]DPA-714 plasma metabolism in WT and Tg 10-month-old rats over 60 min. Blood samples were collected at four time points per animal. The Tg (*t*½ = 23.10 min) and WT rats (*t*½ = 26.66 min) had similar patterns of metabolism. *DUR* differential uptake ratio = % injected dose normalized to animal weight and plasma volume)

The Tg (*n* = 3, 10 ± 1 months) and age-matched WT rats (*n* = 3, 10 ± 0.5 months) were anesthetized with 2–2.5 % isoflurane air/oxygen mixture and injected with 1–2 mCi (37–74 MBq) of [18F]DPA-714 (1.65 ± 0.43 mCi; 61.05 ± 15.91 MBq). Aliquots of blood (400 μl) were taken from each animal at 5, 15, 30, and 60 min post-injection and centrifuged at 17,000×*g* at 4 °C for 4 min. One hundred microliter of plasma was retrieved, and extraction was performed using 200-μl acetonitrile. The mixture was vortexed for 30 s and centrifuged at 17,000×*g*, 4 °C for 4 min. The supernatant was removed and 25 μl from each sample was applied on silica gel thin layer chromatography (TLC) plates. A mixture of chloroform (90%), methanol (9%), and ammonium hydroxide (1%) was used as the eluent. The dried TLC plates were placed overnight on a phosphorimaging plate with a pixel size of 25 μm (Fuji BAS-SR2025, Fujifilm, Japan). After exposure, the plates were scanned using a Fuji FLA-5100 and the data was analyzed using Image Gauge (Fujifilm, Japan). The radioactivity of the remaining plasma and pellets was determined by γ-counting (Perkin Elmer 2480 Wizard3) and used to calculate the percent parent uptake of the plasma, corrected for metabolites (differential uptake ratio, DUR = % injected dose normalized to animal weight and plasma volume). Finally, the percentages of unchanged [18F]DPA-714 in plasma as a function of time were fitted to an exponential decay equation (Fig. 1).

### [^18^F]DPA-714 PET

The 3-month-old group consisted of four Tg (237 ± 46 g) and four age-matched WT rats (263 ± 27 g). The 9-month-old group consisted of five Tg (344 ± 36 g) and five WT rats (410 ± 41 g). The 16-month-old group consisted of six Tg (392 ± 25 g) and three WT rats (464 ± 11 g).

Two to three rodents were scanned per day, and the order of scanning was counterbalanced: one Tg rat and one WT rat, in alternate order. The animals were anesthetized with 2–2.5 % isoflurane air/oxygen mixture. The intra-subject variability of the depth of anesthesia was monitored by measuring respiratory frequency periodically during the scan. The PET experiments were performed on a Bio PET/CT tomograph (Bioscan Inc., Washington, DC) with an axial field of view of 4.8 and 6.7 cm in diameter. Time coincidence window was set to 10 ns with an energy window of 250–700 keV. The lateral tail vein was cannulated for injection of radiotracer, and the cannula was then connected to a heparin lock and secured in place with medical tape. The animal was positioned prone with the head placed symmetrically in the center field of view (FOV) on the thermostatically heated bed supplied by the manufacturer (Bioscan Inc., Washington, DC).

[^18^F]DPA-714 injection of 1.66 ± 0.22 mCi (61.4 ± 8.1 MBq; 2.0 ± 0.9 nmol/rat) was then administered as a bolus injection (30 s) into the indwelling intravenous catheter followed by a 300-μl saline flush (maximum volume of injection = 600 μl). PET emission data was acquired for 60 min in list mode and then reframed into a dynamic sequence of 20 frames (6 × 20, 3 × 60, 11 × 300). The resultant emission Sinogram for each frame were then corrected for attenuation, scatter, ^18^F decay, randoms, and dead time. Dynamic images were reconstructed using OSEM-2D algorithm into 175 × 175 × 61 slices with a voxel size of 0.39 × 0.39 × 0.78 mm. The reconstructed images were co-registered to a template MR in stereotaxic space using a rigid body transformation model as previously described [27]. Time-activity curves were generated for volumes of interest (VOIs) that were drawn manually on the template MR image guided by an anatomical atlas of the rodent brain [28]. The VOI positions were evaluated by displaying the corresponding VOI on the co-registered PET images. Image analyses were performed using PMOD 3.4 kinetic modeling tool (PMOD Technologies Ltd., Zurich, Switzerland). Regional [^18^F]DPA-714 uptake was quantified as standardized uptake value equivalent to percentage of injected dose per cubic centimeter after correction for body weight (SUVc = % ID kg/cm^3^). The SUVs were calculated from the imaging frames obtained between 40 and 60 min after injection. Those frames were assumed to reflect “pseudo equilibrium” status since they had the lowest rate of change in the concentration activity curve (<5 %/h) [29]. The body weight was adjusted according to Kleiber laws [30] as previously described [31] to account for weight differences between our adult Tg and adult WT rat. For the QA injection group, we calculated the ratio of uptake in the ipsilateral striatum (site of injection) and cortex (needle track) to the contralateral striatum and cortex (reference region).

### Immunofluorescence

#### Tissue preparation

The Tg rats (*n* = 14) and WT rats (*n* = 12), ranging in age from 1 to 10 months, were first anesthetized with isoflurane (3 % with 700 cc/min O_2_). This was followed by transcardial perfusion using 100 ml of normal saline (pH = 7.4) and 350 ml of freshly prepared and filtered (0.45-μm filter) 4 % paraformaldehyde (pH 7.4). The brains were removed and post-fixed overnight in 4 % paraformaldehyde at 4 °C followed by three 1-h washes in normal saline at 4 °C. The brains were next cryoprotected in 10 % sucrose and stored at 4 °C until they sank in the solution; they were subsequently placed in 20 % and then 30 % sucrose until they sank again in each solution. The brains were then embedded in optimal cutting temperature compound (OCT, Tissue-Tek®), and 10-μm-thick coronal serial sections were obtained. The striatal sections (bregma 0.48 to 0.12 mm) were then selected for immunofluorescent staining. Immunolabeling protocols were applied to identify the microglial phenotypes in fresh frozen brain slices using rabbit IgG anti-Iba1 (Wako Industries, cat# 019-19741) to identify microglia/macrophages. The above primary immunoreaction was visualized using appropriate fluorophore-conjugated (Alexa Fluor dye) secondary antibodies. The cell nuclei were counterstained using 1 ug/ml DAPI to facilitate cell counting. All fluorescence signals were imaged using an Axio Imager.Z2 upright scanning wide-field fluorescence microscope (Zeiss) equipped with an Orca Flash 4.0 high-resolution sCMOS camera (Hamamatsu), 200W X-cite 200DC broadband light source (Lumen Dynamics), and standard DAPI and Alexa Fluor filter sets (Semrock). After imaging, the image datasets were processed for image stitching and illumination correction and the images were imported into Adobe Photoshop CS6 to produce pseudo-colored composites.

#### Quantification

Quantification of Iba1 immunofluorescent staining was performed using FIJI image processing package, based on ImageJ (NIH, Bethesda, MD). The locations of the selected striatal, hippocampal, and cortical ROIs were identical between all the animals. The RGB bitmap images were first converted to 8-bit grayscale, and the threshold was adjusted to include only cells of interest and eliminate the background. This was followed by counting using the image-based tool for counting nuclei plug-in (ITCN). All images were processed using the same analysis parameters. The Iba1 cell density (cells/mm^2^) was calculated from the total number of positive cells divided by the total area.

### Cytokine/chemokine level measurements in Tg and WT animal brain lysates

#### Brain lysate preparation

Cytokine/chemokine levels were measured in brain lysate solutions from two age groups, 3 month-old (five Tg and five WT) and 9 month-old (five Tg and four WT). Before sacrifice, the animals were perfused with normal saline and then were decapitated. Their brains were excised, placed into a brain matrix (World Precision Instruments, Sarasota, FL), cut into coronal section (3 mm each) and immediately frozen on dry ice then stored in −80 °c. For total protein extraction, 100-mg brain tissue sections were homogenized in 2-ml tissue extraction buffer (Novateinbio, Woburn, MA) containing protease inhibitors (Sigma, St. Louis, MO) at 1:10 dilution. After centrifugation at 19,000×*g* for 20 min at 4 °C, the supernatant was collected and stored in −80 °C until the assay was performed. Total protein concentration from the supernatant was determined using BCA protein assay kit (Pierce, Rockford, IL, USA).

Tissue samples from the striatal and hippocampal areas were processed for each animal, and the samples were run in triplicates. Additional tissue samples from the cerebellum were also processed and run in duplicates. Cytokine/chemokine levels of IL-1α, IL-1β, IL-2, IL-4, IL-5, IL-6, IL-7, IL-10, IL-12, IL-13, IL-17, IL-18, EPO, G-CSF, GM-CSF, M-CSF, GRO/KC, MIP1α, MIP3α, RANTES, IFN-γ, VEGF, TNF-α, and MCP-1 were then determined using a Bio-Plex Pro™ Rat Cytokine 24-plex Assay (Bio-Rad, Hercules, CA, USA) according to the manufacturer’s instructions. Concentrations of cytokine/chemokines were read on the Bio-Plex 200 System (Bio-Rad, Hercules, CA, USA).

### Statistical analysis

All data are represented as mean ± SD. Statistical significance was determined using GraphPad InStat statistical software (version 3.0, San Diego, CA, USA). For the PET scans and immunofluorescent Iba1 cell density values, the differences were compared using two-sample Student’s *t* tests.

For the cytokine/chemokine data, multiple comparisons were performed using non-parametric ANOVA (Kruskal-Wallis) test followed by Dunn’s post hoc analysis. Simple comparisons were made using unpaired two-tailed Student’s *t* test for parametric data (with Welch correction) or Mann-Whitney *U* test for unpaired non-parametric data. A *p* value of <0.05 was considered significant.

## Results

### Animal weight

There were significant differences in body weight between the Tg and age-matched WT rats in the 9-(*p* < 0.05) and 16-month-old (*p* < 0.01) groups; no differences in body weight were observed in the 3-month-old group. Due to the weight differences in the adult rats, we corrected the animal weight using the Kleiber method in which the metabolic activity is proportional to *m*^0.74^ with *m* being the animal’s body weight (in grams) [30]. We chose this method as a compromise between the total body weight method of correction that could underestimate the SUV in the brain and the lean body mass method of correction that could overestimate the SUV in the brain. This method has been used in another similar paper [31].

### Radiochemistry

The chemical purity of our radiotracer was consistently >95%. There were no significant differences in specific activity (SA) values nor in the injected dose (ID) of [^18^F]DPA-714 in the 3-month-old (SA, 1.5 ± 0.6 and 1.3 ± 0.4 Ci/μmol; ID, 1.5 ± 0.3 mCi (55.5 ± 11 MBq) and 1.7 ± 0.2 mCi (62.9 ± 7.4 MBq), respectively), 9-month-old (SA, 2.1 ± 1.1 and 2.1 ± 0.9 Ci/μmol; ID, 1.9 ± 0.9 mCi (70.3 ± 33.3 MBq) and 1.9 ± 1.0 mCi (70.3 ± 37 MBq), respectively), or the 16-month-old Tg and age-matched WT rats (SA, 1.4 ± 0.5 and 1.4 ± 0.6 Ci/μmol; ID. 1.7 ± 0.1 mCi (62.9 ± 3.7 MBq) and 1.6 ± 0.1 mCi (59.2 ± 3.7 MBq), respectively). In the QA-lesioned rats, the SA was 1.6 ± 0.1 Ci/μmol and ID was 1.3 ± 0.4 mCi (48.1 ± 14.8 MBq).

### [^18^F]DPA-714 blood metabolism

No appreciable differences in blood metabolism patterns were observed between the Tg and WT rats. The parent molecule levels were similar in the Tg and WT rats at different time points: 92.40 ± 3.24 % compared to 95.81 ± 1.72 % at 5 min, 61.15 ± 22.49 % compared to 63.37 ± 15.52 % at 15 min, 35.74 ± 11.92 % compared to 37.14 ± 8.00 % at 30 min and 20.31 ± 4.97 % compared to 25.79 ± 6.70 % at 60 min (Fig. 1). Our metabolism results were very similar to previously reported values in the literature [32].

### [^18^F]DPA-714 PET

**Fig. 2:**
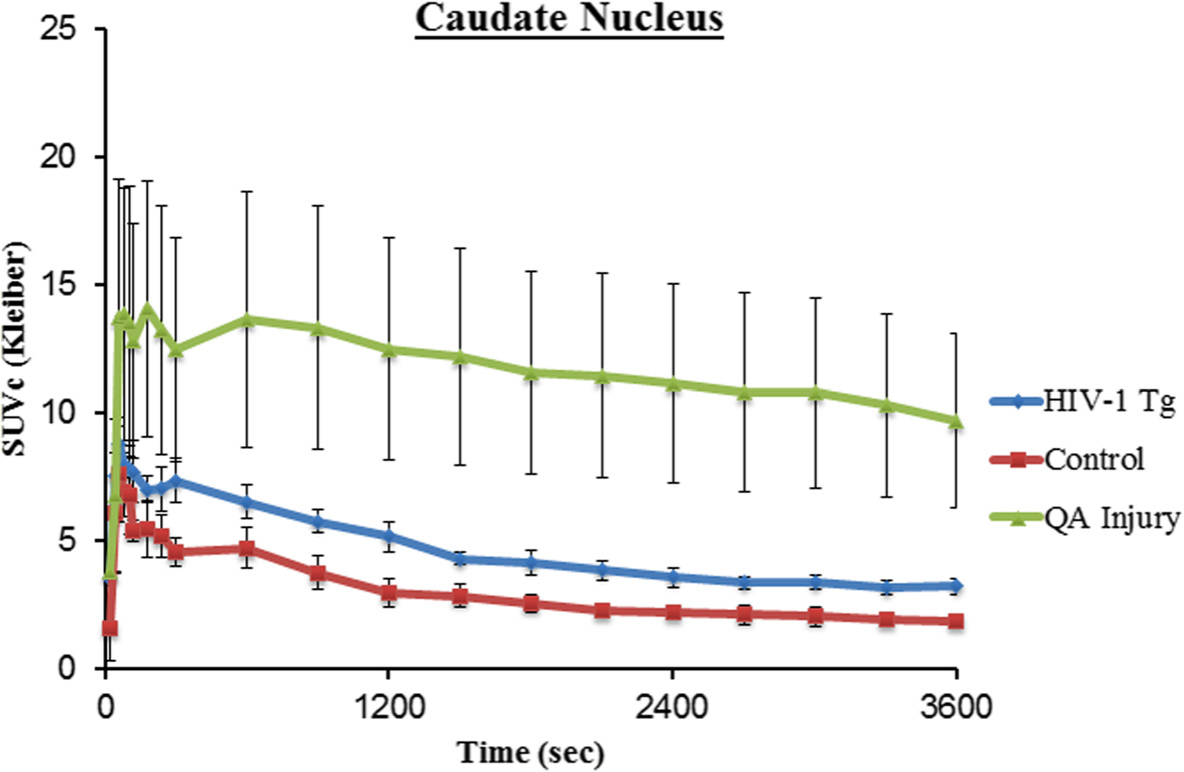
[^18^F]DPA-714 PET time-activity curves over 60 min derived from the caudate nucleus of a 9-month-old Tg, 9-month-old WT rat, and one QA unilaterally lesioned rat. *Error bars* represent standard error of the mean at each time point

The time-activity curves (TACs) of the 60-min acquisitions were similar in shape between the Tg and WT rats in all regions. Representative TACs derived from the caudate nucleus region are shown in Fig. 2 and Additional file 1. The radioligand was rapidly taken up in the brain but displayed fast washout with only small activity concentrations measured at later time points. Pseudo equilibrium was reached at approximately 40 min after injection (Fig. 2).

**Fig. 3:**
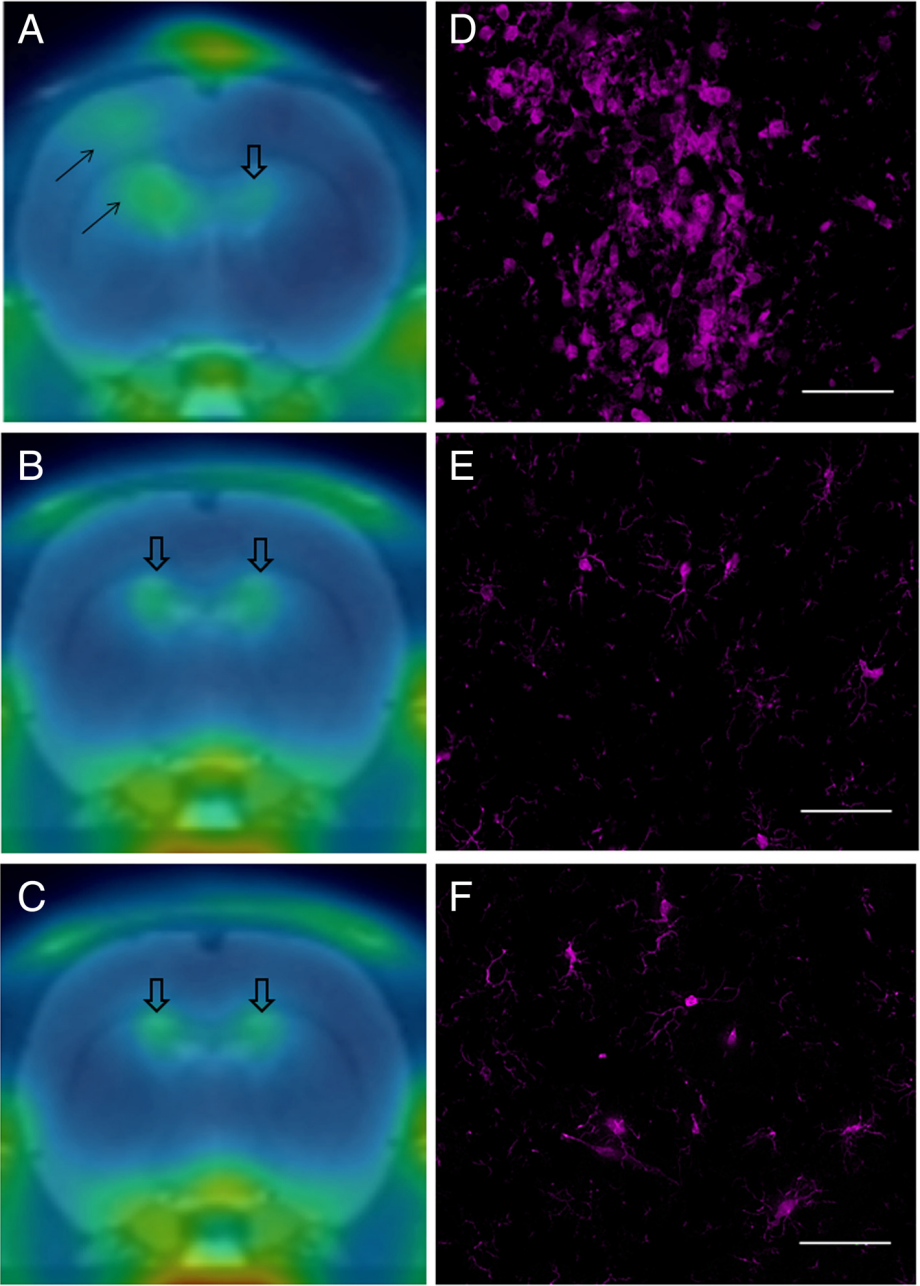
Representative coronal [^18^F]DPA-714 PET summed images between 40 and 60 min of acquisition in **a** QA-injected rat, **b** 9-month-old Tg, and **c** 9-month-old WT rat, in template MRI stereotaxic space. Increased [^18^F]DPA-714 uptake is seen ipsilateral to the QA injury site in **a** (*solid black arrows*). No qualitative differences in uptake between the Tg and WT rats are seen. Areas of uptake in **a**, **b**, and **c** (*open arrows*) are due to normal [^18^F]DPA-714 PET uptake in the choroid plexus within the ventricular system. Intense Iba1 immunofluorescent staining (increased size and number of activated microglia) in the QA-lesioned rat (**d**), with comparable staining of cells in middle-aged Tg (**e**) and age-matched WT (**f**) rats (*scale bar* 100 μm)

In all the three age groups, the Tg rats generally showed slightly increased SUV values compared to the age-matched WT rats; however, this was not statistically significant in any group or any brain region (Table 1, Fig. 3b, c). In the QA-injected rats, on the other hand, there was a definite increase in [^18^F]DPA-714 binding ipsilateral to the injection site when compared to the contralateral side (Fig. 3a). The increased uptake was seen in the caudate at the site of the injection as well as along the tract of the needle in the cortex. The fold increase ranged from 1.2 to 2 (ipsilateral versus contralateral ratio).

**Table 1:** Corrected standardized uptake values (SUVc = % ID. kg/cm^3^) for Tg and age-matched WT rats at three age groups

Region	3-month-old			9-month-old			16-month-old		
	Tg	WT	*p* value	Tg	WT	*p* value	Tg	WT	*p* value
Cortex	3.07 ± 0.23	2.87 ± 0.79	0.65	2.81 ± 0.71	2.32 ± 0.42	0.21	2.19 ± 0.43	1.61 ± 0.29	0.1
Caudate	2.62 ± 0.18	2.68 ± 0.87	0.90	2.60 ± 0.62	2.02 ± 0.41	0.11	2.18 ± 0.53	1.58 ± 0.22	0.11
Thalamus	2.32 ± 0.27	2.12 ± 0.45	0.47	2.51 ± 0.66	2.06 ± 0.38	0.22	1.98 ± 0.50	1.42 ± 0.14	0.11
Hippocampus	2.35 ± 0.38	2.14 ± 0.47	0.50	3.41 ± 0.60	2.54 ± 0.79	0.1	1.93 ± 0.63	1.40 ± 0.17	0.21
Cerebellum	3.40 ± 0.17	3.14 ± 0.80	0.54	3.37 ± 1.00	2.94 ± 0.62	0.42	2.60 ± 0.71	1.90 ± 0.22	0.15

Values are listed as mean SUVc ± SD for each group

### Immunofluorescence

The Iba1 cell density values (cells/mm^2^) were not significantly different between the Tg and WT rats in any of the evaluated brain regions, which included the cortex, striatum, and hippocampus (two-sample Student’s *t* test, *p* > 0.05).

### Cytokine/chemokine measurements in the brain lysates

**Fig. 4:**
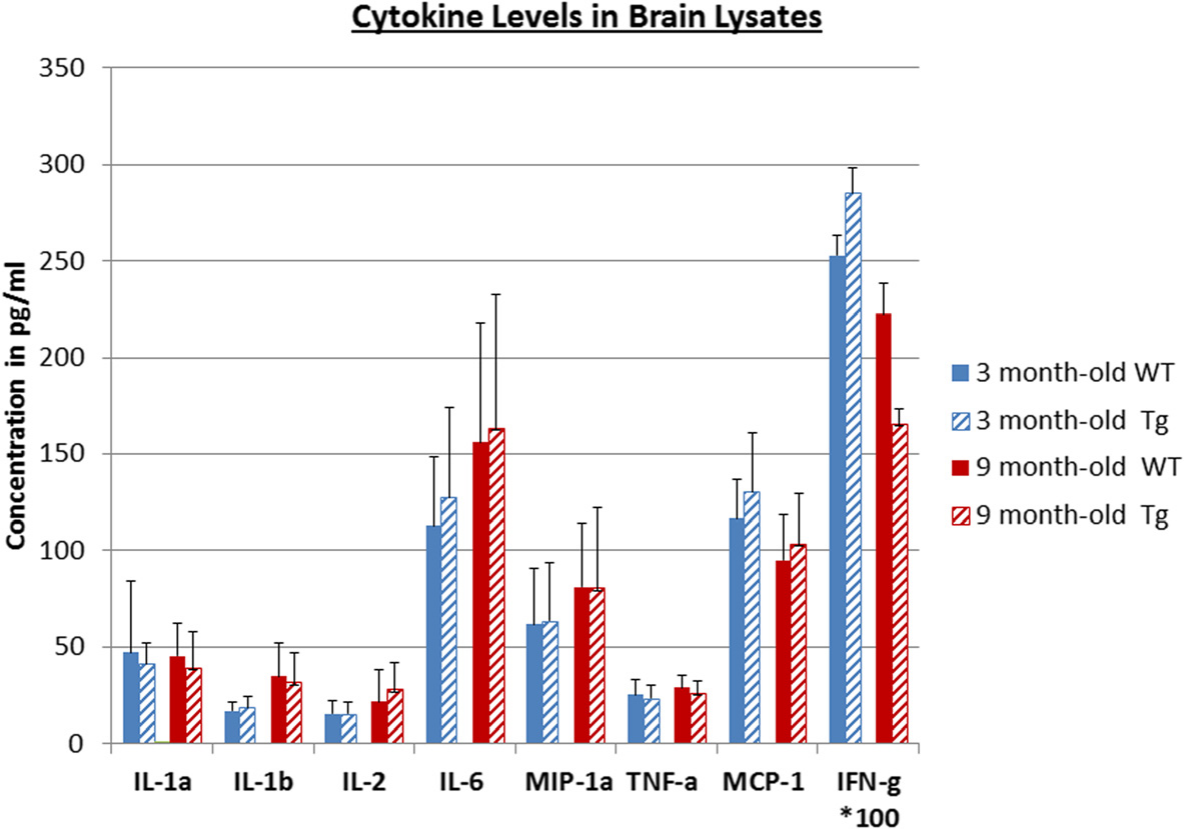
Brain lysate concentrations of select cytokine/chemokines in Tg and WT rats, at two age groups (3 and 9 months old). There were no statistically significant differences in cytokine/chemokine levels between the Tg and WT animals at any age

For all the measured cytokine/chemokines in the brain lysates, there were no significant differences in cytokine/chemokine concentrations between the Tg and age-matched WT animals at any age (Fig. 4).

When we compared the general direction of change with age between the TG and WT rats, there were no appreciable differences with cytokine/chemokine concentrations generally following the same pattern of either increase, decrease, or no change in both groups.

## Discussion

The exact neuropathology leading to milder forms of HIV-associated neurocognitive disorders (HAND) is not fully understood. Unlike microglia and astrocytes, neurons do not express CD4 and there is no direct evidence of productively infected neurons with HIV [33, 34]. The neurologic damage is rather thought related to persistent low level of neuroinflammation [5, 35], neurotoxic effects of viral proteins [36, 37], as well as the indirect disruption of the supportive and neurotrophic role of astrocytes [38] and oligodendrocytes [39]. Among those factors, the contribution of neuroinflammation (microglial activation) to neuronal damage is believed to play a major role [8, 5, 6, 4, 7, 3]. As a result, there were three attempts to image microglial activation in vivo in HIV-positive (HIV+) patients using PET. All three groups [40–42] used [^11^C]PK11195; however, the results were inconsistent, which is probably due to the heterogeneity of patient populations, as far as treatment and neurological status, as well as the limitations of [^11^C]PK11195 as a radiotracer [17–20].

One way of controlling for those variables is to perform imaging studies in animal models. The Tg rat is a non-infectious small animal model of HIV infection, in which the expression of the transgene, consisting of an HIV-1 provirus with functional deletion of gag and pol, is regulated by the viral long terminal repeat [43]. There is no potential for replication, but there is chronic exposure to viral proteins, such as Tat, gp120, Vpr, and Nef. The HIV-1 Tg rat exhibits pathologies and immune irregularities characteristic of HIV-1 infection of humans. As a model for HIV infection, it was found to develop consistent neurological and behavioral deficits [44–47], increased expression of microglial markers such as CD11b, and, with some groups [48, 35, 49], but not others [50], increased levels of multiple cytokines and chemokines. It thus appears to be of particular importance for the evaluation of neurological complications of HIV. The non-infectious nature of the model, the larger brain size allowing for microPET imaging (compared to mice) and the commercial availability of the Tg rat are additional positive factors. In our study, we set out to validate [^18^F]DPA-714 as an in vivo biomarker of microglial activation in the Tg rat so that we can ultimately use the combination of the animal model and ligand in the evaluation of the effectiveness of neuroprotective therapies/approaches.

[^18^F]DPA-714 has been useful in the study of other animal models of neuroinflammation including encephalitis [18], cerebral ischemia [20, 51], epilepsy [52], excitotoxicity [53], glioma [32, 54, 55] and other preclinical neurodegenerative disease models. Useful neuroimaging probes should fulfill a number of key general properties: appropriate lipophilicity, lack of toxicity, small size with high selectivity for the target site, and preferably lack of radiolabeled metabolites that can cross the blood brain barrier. With PET ligands, however, differences in cerebral metabolism have been described in genetically engineered small animal models and/or pathologically modified rodents [56]. Therefore, since we are dealing with a transgenic animal model, we evaluated plasma [^18^F]DPA-714 metabolism and found no difference between the Tg and WT rats, with similar clearance rates of the ligand from the blood in both groups.

We chose to evaluate three different age groups (young, middle-aged, and old) because we assumed the older animals would demonstrate more neuroinflammation by virtue of longer exposure to viral proteins. We also based our choice on other functional and structural abnormalities that we previously detected in the Tg rats at those ages and that worsened with aging [57, 58]. Using [^18^F]DPA-714, we found higher mean SUVc values in the Tg rats compared to the age-matched WT rats; however, this difference did not reach statistical significance in any region or age group. In the unilateral QA striatal injection model (known to induce inflammation and increased TSPO expression [19]), on the other hand, there was clear increased uptake in the ipsilateral striatum (Fig. 3a). Immunofluorescent staining confirmed the *in vivo* findings with no qualitatively appreciable difference in Iba1 cellular counts in the Tg brains compared to age-matched WT rats (Fig. 3e, f). Microglial activation on the other hand was clearly demonstrated in the QA-lesioned rat brains, ipsilateral to the injection site/needle tract (Fig. 3d).

To further support our findings, we measured the concentrations of 24 cytokine/chemokines in the brain lysates of the Tg and WT animals at two different ages: 3 and 9 months. This was of relevance since the literature was not consistent about this topic: while some groups detected increased cytokine/chemokines [35, 59], others did not [50]. In our hands, there were no significant differences in cytokine/chemokine concentrations in brain lysates between the Tg and WT rats at either age (Fig. 4).

One limitation of our study is that direct estimation of specific binding was not possible since there is no suitable reference region available for TSPO binding in this model with diffuse neuropathology, and arterial blood sampling is not logistically feasible. In such cases, it is not unusual to perform qualitative comparison of the regional distribution of the tracer (SUV) in the different groups of animals if the studies are run in parallel. In fact, other researchers have previously used SUV quantification with [^18^F]DPA-714, with success [54, 60–62]. We were also encouraged to use SUV by a recent paper that found significant correlation between SUV values and distribution volume measurements in rat brains using a similar TSPO ligand [63]. Unfortunately, a similar comparison has not been done using [^18^F]DPA-714 in rats. Another inherent limitation of the model of our study is the discrepancy in weight between the Tg and WT animals as they grow older. To minimize the effect associated with this discrepancy, we decided to correct SUV values for weight differences [30, 31].

In conclusion, even though the HIV-1Tg rat is a good animal model of viral protein neurotoxicity and treated HIV+ patients [46, 49, 59, 64, 65], we did not find appreciable microglial activation to allow the use of [^18^F]DPA-714 PET imaging as a biomarker of neurodegeneration in the evaluation of existing and emerging neuroprotective therapies. Alternative imaging targets relevant to the neuropathology of the Tg rat should thus be sought, such as those related to oxidative stress [66–69], arachidonic acid metabolism [48], NMDA excitotoxicity, abnormal dopamine receptor signaling/tyrosine metabolism [57, 70], abnormal myelination [70], and astrocytic death [58].

## Additional file

Additional file 1: Figure S1. [^18^F]DPA-714 PET time-activity curves over 60 min derived from the caudate and hippocampus of 3-, 6-, and 9-month-old Tg and WT rats. *Error bars* represent standard deviation values at each time point. (TIFF 2460 kb)

## Abbreviations

ACUC: Animal Care and Use Committee
ART: antiretroviral therapy
HAND: HIV-associated neurocognitive disorders
ID: injected dose
NIH: National Institutes of Health
PET: positron emission tomography
QA: quinolinic acid
SA: specific activity
Tg: HIV-1 transgenic rat
TLC: thin layer chromatography
TSPO: translocator protein
WT: wild type
