# Supplementary figures and images for: Lack of neuroinflammation in the HIV-1 transgenic rat: an [18F]-DPA714 PET imaging study
Source: J Neuroinflammation. 2015 Sep 17;12:171. doi: 10.1186/s12974-015-0390-9 (PMC4574011; doi:10.1186/s12974-015-0390-9)

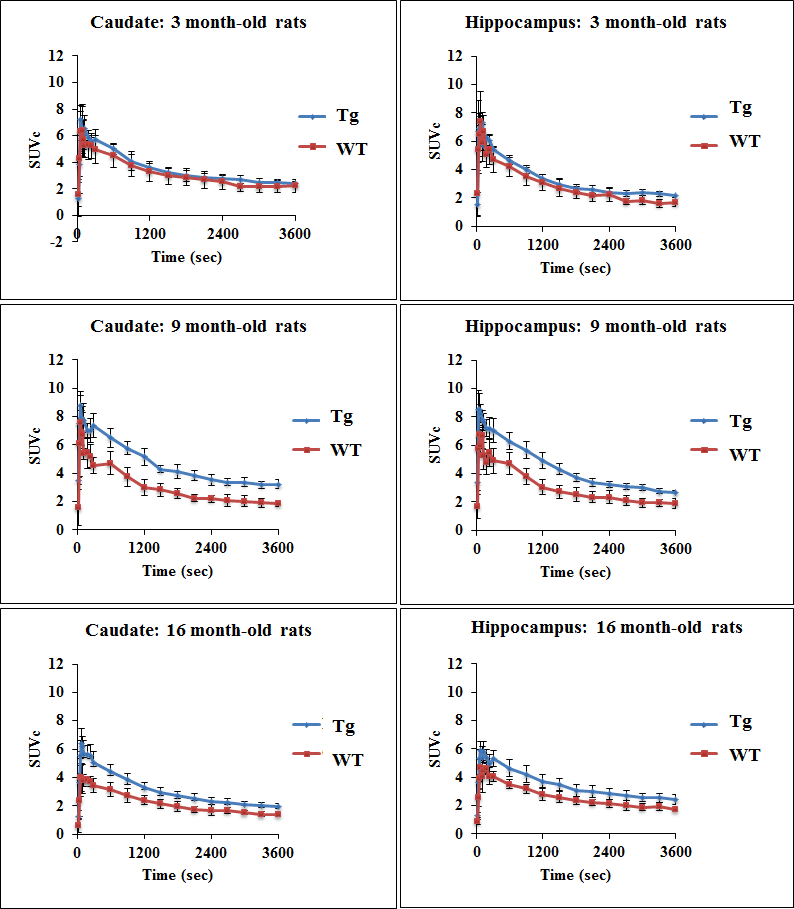

Supplement: Additional file 1: Figure S1. — [18F]DPA-714 PET time-activity curves over 60 min derived from the caudate and hippocampus of 3-, 6-, and 9-month-old Tg and WT rats. Error bars represent standard deviation values at each time point. (TIFF 2460 kb) [file 12974_2015_390_MOESM1_ESM.tif]
